# Intraoperative use of enriched collagen and elastin matrices with freshly isolated adipose-derived stem/stromal cells: a potential clinical approach for soft tissue reconstruction

**DOI:** 10.1186/1471-2482-14-10

**Published:** 2014-02-20

**Authors:** Ziyad Alharbi, Sultan Almakadi, Christian Opländer, Michael Vogt, Hans-Oliver Rennekampff, Norbert Pallua

**Affiliations:** 1 Department of Plastic, Reconstructive and Hand Surgery - Burn Center, Medical Faculty, RWTH Aachen University, Pauwelsstr. 30, Aachen D-52074, Germany; 2 Division of Plastic Surgery, Specialist Surgery Center, King Abdullah Medical City, Mecca, Kingdom of Saudi Arabia; 3 Two-Photon Microscopy Facility, Interdisciplinary Center for Clinical Research (IZKF), Medical Faculty, RWTH Aachen University, Aachen, Germany

**Keywords:** Adipose tissue-derived stem/stromal cells, Stromal vascular fraction, Liposuction, Fat grafting, Biomaterials, Collagen-based scaffolds, Regeneration and tissue engineering

## Abstract

**Background:**

Adipose tissue contains a large number of multipotent cells, which are essential for stem cell-based therapies. The combination of this therapy with suitable commercial clinically used matrices, such as collagen and elastin matrices (i.e. dermal matrices), is a promising approach for soft tissue reconstruction. We previously demonstrated that the liposuction method affects the adherence behaviour of freshly isolated adipose-derived stem/stromal cells (ASCs) on collagen and elastin matrices. However, it remains unclear whether freshly isolated and uncultured ASCs could be directly transferred to matrices during a single transplantation operation without additional cell culture steps.

**Methods:**

After each fat harvesting procedure, ASCs were isolated and directly seeded onto collagen and elastin matrices. Different time intervals (i.e. 1, 3 and 24 h) were investigated to determine the time interval needed for cellular attachment to the collagen and elastin matrices. Resazurin-based vitality assays were performed after seeding the cells onto the collagen and elastin matrices. In addition, the adhesion and migration of ASCs on the collagen and elastin matrices were visualised using histology and two-photon microscopy.

**Results:**

A time-dependent increase in the number of viable ASCs attached to the collagen and elastin matrices was observed. This finding was supported by mitochondrial activity and histology results. Importantly, the ASCs attached and adhered to the collagen and elastin matrices after only 1 h of *ex vivo* enrichment. This finding was also supported by two-photon microscopy, which revealed the presence and attachment of viable cells on the upper layer of the construct.

**Conclusion:**

Freshly isolated uncultured ASCs can be safely seeded onto collagen and elastin matrices for *ex vivo* cellular enrichment of these constructs after liposuction. Although we observed a significant number of seeded cells on the matrices after a 3-h enrichment time, we also observed an adequate number of isolated cells after a 1-h enrichment time. However, this approach must be optimised for clinical use. Thus, *in vivo* studies and clinical trials are needed to investigate the feasibility of this approach.

## Background

Adipose tissue stores a large number of multipotent cells, which are essential for stem cell-based therapies [1]. Stem and progenitor cells typically comprise approximately 3 to 7% of the cells in the uncultured stromal vascular fraction (SVF) from adipose tissue [1,2]. The term SVF is commonly used in the literature and refers to the cellular pellet without fat cells (i.e., mature adipocytes). This fraction can be obtained through an isolation process that uses collagenase secondary to liposuction (i.e., fat harvesting), resulting in a component that contains multiple cells: adipose-derived stem/stromal cells (ASCs). ASCs consist of endothelial cells, smooth muscle cells, fibroblasts and stem cells. [2]. Adipose-derived stem cells are adult self-renewing cells of mesenchymal origin that can differentiate not only into the adipogenic lineage but also into the osteogenic, chondrogenic, myogenic and neurogenic lineages *in vitro*[3,4].

We previously demonstrated that the method of liposuction influences the viability of the ASCs isolated from adipose tissue and the quantity of growth factors produced after cellular transplantation onto clinically used collagen and elastin matrices [5]. The results also indicated that collagen and elastin matrices could be used as *ex vivo* cellular carriers for tissue engineering*.* In this context, several studies investigated the safety of collagen and elastin matrices for keratinocyte and ASC cultivation [6]. Tissue engineering protocols that include a specific time period for the cultivation of ASCs require the use of a lab facility for as long as several days. Such protocols require Good Manufacturing Practicing (GMP) facilities and may be expensive. In contrast, the manual or automatic isolation of ASCs can be performed directly in the operating room (e.g., using a Cytori device). Thus, the direct transfer of ASCs onto available commercial matrices would be possible during a single operation. However, a short cultivation time (i.e., 1 to 3 hours) would be ideal for transplantation, particularly in a clinical setting with no access to further lab processing or GMP facilities.

**Figure 1:**
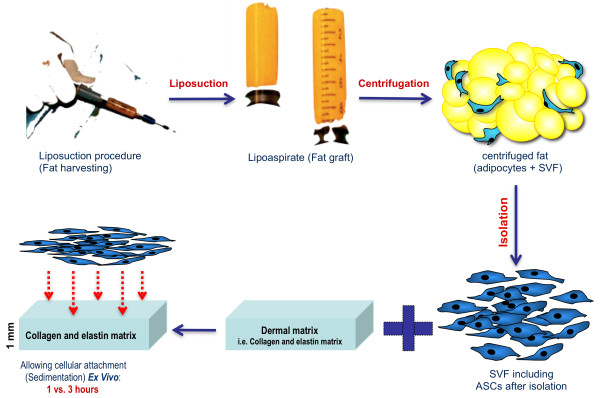
**An illustration of the described approach.** After isolation of cells from adipose tissue secondary to liposuction, the obtained adipose-derived stem/stromal cells were directly transferred to the collagen and elastin matrix without additional cell culture steps. The cells were incubated on top of the material for different clinically relevant time periods (i.e., 1 and 3 h) for cellular matrix enrichment. For comparison, the cells were incubated with the matrices for 24 h.

The time required for *ex vivo* attachment of adipose-derived cells to collagen and elastin matrices before transplantation of the enriched construct to the patient during soft tissue reconstruction remains unknown. The results obtained in our previous study demonstrated that a significant number of cells attached to the collagen and elastin matrices after 24 hours of cultivation, regardless of the method of liposuction. However, this time is not realistic for a surgeon performing surgery under anaesthesia. Therefore, we compared the enrichment of collagen and elastin matrices with ASCs after short time periods (i.e., 1 h and 3 h) and long time periods (i.e., 24 h) to define a protocol that avoids delays in the operating room (Figure 1).

These time intervals were chosen to determine whether a 3-h time period is needed for cellular sedimentation onto the scaffolds or whether a 1-h sedimentation time is adequate for the cells to attach and migrate onto the collagen and elastin matrices prior to transplantation of the enriched construct in the operating room. We compared the results obtained using short cultivation times (1 and 3 h) with the results obtained using a 24-h cellular cultivation time, which was demonstrated to be a successful approach in our previous study [5]. We designed the experiment without the need for processing of the cells to simulate clinical conditions. Thus, for example, a patient could have a single operation that included the enrichment of the selected collagen-based matrix and split thickness skin transfer for multi-layer reconstruction of soft tissue defects.

## Methods

### Patients

Ten healthy patients (5 males and 5 females) between 27 and 59 years of age had elective liposuction (i.e., fat harvesting) in the Department of Plastic, Reconstructive and Hand Surgery – Burns Center at RWTH Aachen University Hospital. Each patient signed the consent form. The protocol and the use of human material were approved by the ethics committee of the Faculty of Medicine at RWTH University in Aachen, Germany (Name in German: Ethik-Kommission des Universitätsklinikums Aachen, Votum Number: EK163/07). The experiments were conducted in compliance with the Declaration of Helsinki Principles.

### Liposuction and centrifugation of the obtained lipoaspirate

Fat was harvested via tumescent liposuction, as described previously [5,7]. The harvesting cannula used in this study was the st’RIM cannula (Thiebaud Biomedical Devices, Margencel, France), which was developed by Guy Magalon for micro-lipografting. This cannula was 2 mm in diameter with a blunt tip and four 600-μm gauge orifices. After the liposuction procedure, the samples were centrifuged for 3 min at 3,000 rpm using a Sigma 2–16 K centrifuge (Osterode am Harz, Germany). After centrifugation, the purified lipoaspirate was immediately used in experiments (Figure 1).

### Isolation of ASCs from lipoaspirate

Isolation of the cellular pellet was performed as described previously [5]. Briefly, the purified lipoaspirate was transferred into a sterile tube and normal saline was added to remove cell debris and blood. A second centrifugation process was then completed for 10 min at 300 × g. The extracellular matrix was digested with 0.075% collagenase I (Biochrom, Berlin, Germany) for 45 min at 37°C. The digested tissue solution was subsequently filtered using a 250 μm filter (Neolab, Heidelberg, Germany). The pellet was resuspended in 30 ml of a NaCl solution and centrifuged for another 10 min at 300 × g to obtain the SVF, which contained the ASCs. The pellet was then resuspended in DMEM/F12 supplemented with 100 U/ml of penicillin and 100 μg of streptomycin, without foetal calf serum or proliferation factors. The isolated cells were not cultured or passaged prior to direct transplantation onto the collagen and elastin matrix.

### Incubation of ASCs on collagen and elastin matrices to assess cellular adherence

Circular 1 mm thick pieces of non–cross-linked native bovine collagen and elastin matrix containing type I, III, and V collagen derived from bovine skin were used (Matriderm® sheet; MedSkin Solutions Dr. Suwelack AG, Billerbeck, Germany). A circular punch biopsy device measuring 0.8 cm in diameter was used to cut the matrix into small pieces, which were placed into 48-well culture plates. The isolated cells were added to 48-well culture plates lined with collagen and elastin matrix at a density of 50,000 cells per well and incubated at 37°C with 5% CO_2_ for 1, 3 or 24 h. The collagen and elastin matrix that was incubated with the isolated cells was separated after a 1-, 3- or 24-h incubation period (Figure 1). The matrices were washed carefully with normal saline (0.9% NaCl) and transferred into a clean culture Plate. A 270 μl volume of DMEM/F12 supplemented with 100 U/ml penicillin and 100 μg/ml streptomycin was added, and 30 μl of the alamarBlue® resazurin reagent (AbD Serotec, Oxford, UK), was subsequently delivered to each well. This assay can be used to detect the metabolic activity of cells using a fluorescence spectrometer. The medium/alamarBlue® mix was repeated and carefully removed from the well after 2 hours at 37°C and 5% CO_2_. The samples were then measured at room temperature using a Fluostar Optima fluorescence spectrometer (BMG Labtech, Offenburg, Germany), with an excitation wavelength of 540 nm and an emission wavelength 590 nm. We avoided measuring the matrix itself to avoid the influence of the matrix on fluorescence. The alamarBlue® reagent was added to the medium without cells as a negative control.

### Analysis of cellular attachment and migration by histology

Cell-loaded pieces of collagen and elastin matrix were histologically investigated directly after cellular transplantation onto the matrix. The samples were fixed overnight in Lidi’s 4% formalin (Merck, Darmstadt, Germany). The formalin was then removed by extensive washing, and the samples were dehydrated using an increasing gradient of isopropanol, embedded into paraplast, and cut into 15 μm sections. Staining was then performed using hematoxylin and eosin. Microscopic analyses of all samples were performed via light microscopy in our laboratory.

### Analysis of cellular distribution on collagen and elastin matrices using two-photon microscopy

Two-photon microscopy using an FV1000MPE microscope (Olympus Corp., Tokyo, Japan) attached to a pulsed Ti-Sapphire laser (MaiTai DeepSee, SpectraPhysics, Santa Clara, CA, USA) was performed to visualise the 3D-structure of the collagen and elastin matrices, including the organisation of the isolated cells within the matrix. Hoechst 33342 was added for vital staining of the nuclei. The enriched collagen and elastin matrices were also stained with fluorescein diacetate (FDA) to image the isolated cells in combination with Hoechst 33342 for nuclei staining. The enriched collagen and elastin matrix was then visualised using the non-linear optical effect of second harmonic generation (SHG). Hoechst was excited at 730 nm and detected at 418–468 nm. Series of subsequent 1024 × 1024 pixel xy-frames were then obtained in 1 mm z-steps for structural 3D reconstruction using Imaris Software (Bitplane, Zurich, Switzerland).

### Statistical analysis

Data analysis was performed using Prism® software, version 5.01c (GraphPad, La Jolla, CA, USA). A Gaussian distribution of the values was assessed using the D’Agostino & Pearson omnibus normality test. One-way repeated measures ANOVA was performed followed by an appropriate post-hoc multiple comparison test (i.e., Tukey method). Differences for which *p < 0.05* were considered statistically significant.

## Results

### Assessment of ASC adherence to collagen and elastin matrices

**Figure 2:**
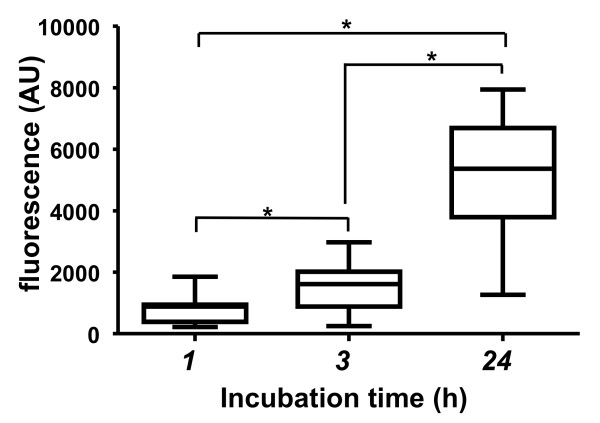
**Adherence of viable adipose-derived cells onto collagen and elastin matrices.** The box-and-whisker plots display the fluorescence signals obtained from adhered and viable adipose-derived cells at different time points (i.e., 1, 3, and 24 h) after seeding on collagen and elastin matrices, as indicated by the alamarBlue resazurin-based assay. The bottom and top of the box represent the first and third quartiles, the band inside the box represents the median, and the whiskers represent the minimum and the maximum of the data. All data values are presented in the results section. Details related to the statistical analysis are presented in the Methods section. n = 10, *p < 0.05, arbitrary unit; AU.

The relative viability and adherence rates of the isolated adipose-derived cells on the collagen and elastin matrices were tested using the alamarBlue-assay® after different enrichment times on the matrices (i.e., 1 h, 3 h, and 24 h). A significantly larger number of viable cells were attached to the collagen and elastin matrices after a 3-h enrichment time (mean: 1,551 and SD: 790; median: 1615 and range: 284.5-2,972) than after a 1-h incubation time (mean: 791.4 and SD: 471.3; median: 882.5 and range: 219.5-1,856) (Figure 2). The observed cellular viability and adhesion rates were 3- to 4-fold higher after a 24-h incubation (mean: 5,120 and SD: 2,070; median: 5,368 and range: 1,267-7,947). However, it should be noted that freshly isolated uncultured ASCs attached and adhered to the collagen and elastin matrices after only 1 h of incubation, indicating that this approach is clinically relevant.

### Analysis of cellular attachment and migration using histology

**Figure 3:**
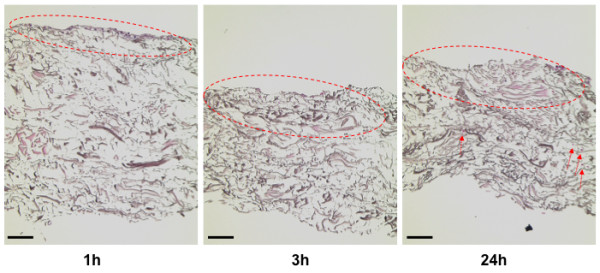
**Histology of enriched collagen and elastin matrices.** This figure shows representative histology images of the collagen and elastin matrices, which were incubated with freshly isolated adipose-derived stem/stromal cells for the indicated time periods (scale bar is 200 μm).

Cell-loaded pieces of collagen and elastin matrix were investigated histologically 1, 3 and 24 hours after seeding the ASCs onto the matrices. After a 1-h incubation, the seeded ASCs were observed on the upper layer of the matrices (Figure 3). Cells that were incubated on the collagen and elastin matrices for 3 h exhibited small cytoplasmic fragments deeper in the matrices. ASCs incubated on the collagen and elastin matrices for 24 hours exhibited the same migration process into the middle layer.

### Visualisation of the 3D structure of collagen and elastin matrices to assess morphology and attachment of seeded ASCs

**Figure 4:**
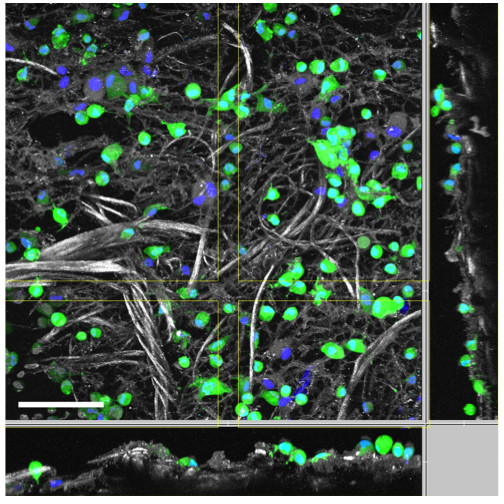
**Two-photon microscopy of the enriched collagen and elastin matrices.** This figure shows a representative two-photon microscopy image of a collagen and elastin matrix, which was incubated with freshly isolated adipose-derived stem/stromal cells for only 1 h. The green colour indicates the cytoplasm of viable cells, which was stained using fluorescein diacetate (FDA), the blue colour indicates the cell nuclei, which were stained using Hoechst 33342, and the grey colour indicates the collagen and elastin structure (scale bar is 100 μm).

The enriched collagen and elastin matrices were investigated after a 1-h enrichment time via two-photon microscopy using autofluorescence to reveal the collagen and elastin structures. Based on the results we obtained using two-photon microscopy with a 24-h time point in our previous study [5], we concentrated on early time points. Viable cells with visible cytoplasm and nuclei were observed on the 3D structure of the collagen and elastin matrix (Figure 4). The previously discussed metabolic activity tests and histological investigations demonstrated that a number of isolated cells attached to the collagen and elastin matrices after 1 h. The green colour indicates the cytoplasmic portion of cells, which was stained with FDA, and the blue colour shows the nuclei, which were stained with Hoechst 33342. The spatial distribution of the cells is also shown (Figure 4).

## Discussion

The clinical applicability of adipose-derived stem cell therapy has been investigated extensively during the last decade, and interesting results have been obtained with respect to regeneration and wound healing, as well as reconstruction and rejuvenation [4]. One advantage of this approach is that adipose tissue represents an easily available and accessible source of mesenchymal stem cells that are supported by abundant stromal cells and growth factors [5]. However, it is essential to determine the applicability of this approach, particularly with respect to legal issues. In some countries, including Germany, any patient materials that leave the operating room for stem cell processing and future re-injection must be processed in GMP facilities. These facilities are expensive and difficult to find. In contrast, autologous transplantation of ASCs in a single session would overcome this problem. Several previous studies investigated the effect of enriching fat grafts with the SVF in a clinical setting [8]. However, it remains unclear whether the enrichment of a safe and biocompatible collagen and elastin matrix with autologous and freshly isolated ASCs is possible. In addition, the role of enrichment time in this approach has not been investigated. Studies addressing these issues could expand the use of soft tissue matrices in combination with ASCs.

Biocompatible matrices, such as collagen and elastin matrices, are currently used in combination with skin grafting as double-layer constructs to support the graft and the formation of a neodermis [9]. However, it must be determined not only whether such matrices are biocompatible but also whether they are cytocompatible. In our previously published study, we demonstrated that freshly isolated uncultured ASCs adhere to collagen and elastin matrices *ex vivo* in only 24 hours. This result indicates that these biocompatible materials are also cytocompatible and appropriate for use with mesenchymal stem cells immediately after their isolation.

However, a 24-h enrichment time is too long to be implemented in the operating room. Thus, a shorter enrichment time of the selected scaffolds with the stem cells is required. We investigated different time intervals to determine whether a 3-h or 1-h cellular sedimentation time on the scaffolds is sufficient to support cellular attachment and migration onto the collagen and elastin matrices prior to transplantation of the enriched construct in the operating room. We compared the results obtained using these short cultivation times (i.e., 1 and 3 h) with the results obtained using a 24 h cellular cultivation time.

In this study, we observed that cellular enrichment on the collagen and elastin matrices after 3 h is improved compared to that observed after 1 h. These results were supported by mitochondrial activity results, which indicate viability, and histological analysis [10-13]. In addition, we demonstrated that enrichment of a collagen and elastin matrix with cells obtained from adipose tissue is possible after a 1-h *ex vivo* enrichment time. This conclusion was also supported by two-photon microscopy results, which revealed the presence and attachment of viable cells on the upper layer of the construct. However, penetration depth is limited based on the employed material, tissue or scaffold (e.g., for collagen and elastin matrices up to 150 μm).

Protocols must be defined for clinical use. Such protocols should include the ideal time for cellular enrichment on the selected biocompatible and cytocompatible matrices, the number of isolated adipose cells per square meter, the proportions of specific cellular elements, including stem cells, in the total population, the commitment of those stem cells toward adipogenic conversion or conversion into other lines *in vivo* and the factors important for control of the environment, including factors that support nutrition and angiogenesis. These factors must be investigated *in animal models* before this type of therapy can be applied in a clinical setting. The fate of ASC-enriched collagen and elastin matrices after transplantation can only be determined *in vivo* in clinical trials. However, prior to clinical trials, it is also necessary to optimise this approach. Specifically, the type of isolated cells that adhere to the matrix must be characterised. The regenerative potential of these cells and the potential benefits for patients should also be addressed in future studies. As previously discussed, the use of the freshly isolated uncultured ASCs seeded directly onto biocompatible scaffolds during a single surgery avoids the need for GMP facilities. Thus, this approach would bridge the gap between the bench and the bedside.

## Conclusion

Freshly isolated uncultured ASCs can be safely seeded onto collagen and elastin matrices after a liposuction procedure for *ex vivo* cellular enrichment of these constructs. Although we observed a significant number of transplanted cells on the matrices after a 3-h enrichment time, we could also observed an adequate number of isolated cells on the matrices after a shorter 1-h enrichment time. This finding was also supported by two-photon microscopy. However, it is necessary to optimise this approach for clinical use. Thus, *in vivo* studies and clinical trials are needed to investigate the feasibility of this approach.

## Competing interests

The authors declare that they have no competing interests.

## Authors’ contributions

ZA drafted the manuscript, participated in study design and conducted the isolation procedure, in vitro procedures and helped in statistical analysis. CO performed the statistical analysis and assisted in histology. HR participated in the coordination of the study and helped to draft the manuscript. SA aided in the organisation of materials and assisted during surgery. MV performed the two-photon microscopy. NP participated in the study design, performed all liposuction procedures, funded this study and helped to draft the manuscript. All authors read and approved the final manuscript.

## Pre-publication history

The pre-publication history for this paper can be accessed here:

http://www.biomedcentral.com/1471-2482/14/10/prepub
